# Telomere length shortening in hospitalized preterm infants: A pilot study

**DOI:** 10.1371/journal.pone.0243468

**Published:** 2021-01-20

**Authors:** Mandy Brown Belfort, Farah Qureshi, Jonathan Litt, Michelle Bosquet Enlow, Immaculata De Vivo, Katherine Gregory, Henning Tiemeier

**Affiliations:** 1 Department of Pediatric Newborn Medicine, Brigham and Women’s Hospital, Boston, Massachusetts, United States of America; 2 Harvard Medical School, Boston, Massachusetts, United States of America; 3 Department of Social and Behavioral Sciences, Harvard T.H. Chan School of Public Health, Boston, Massachusetts, United States of America; 4 Department of Neonatology, Beth Israel Deaconess Medical Center, Boston, Massachusetts, United States of America; 5 Department of Psychiatry, Boston Children’s Hospital, Boston, Massachusetts, United States of America; 6 Department of Epidemiology, Harvard T.H. Chan School of Public Health, Boston, Massachusetts, United States of America; Centre Hospitalier Universitaire Vaudois, FRANCE

## Abstract

Leukocyte telomere length is a biomarker of aging-related health risks. Hospitalized preterm infants frequently experience elevated oxidative stress and inflammation, both of which contribute to telomere shortening. Our aim was to examine changes in telomere length during neonatal intensive care unit (NICU) hospitalization in a cohort of preterm infants <32 weeks’ gestation. We conducted a longitudinal study of 10 infants (mean gestational age 27 weeks, range 23.5 to 29, at birth). We isolated DNA from dried blood spots and used Real Time Quantitative PCR to measure relative leukocyte telomere length in triplicate at three time points for each participant. From birth to discharge, infants experienced an average decline in relative telomere length of 0.021 units per week (95% CI -0.040, -0.0020; p = 0.03), after adjustment for gestational age at birth. Our results suggest a measurable decline in telomere length during NICU hospitalization. We speculate that telomere length change may convey information about NICU exposures that carry short- and long-term health risks.

## Introduction

Telomeres are repeating nucleotide sequences of variable number that protect against chromosome deterioration and regulate cellular and tissue function [1]. Telomere attrition is a well-established biomarker of aging. In adults, shorter telomere length is associated with earlier mortality [2], impaired cognitive function [3], cardiovascular disease [4,5], and chronic obstructive pulmonary disease [6]. While heritable to some extent [7], the rate of telomere attrition is susceptible to environmental influences, with effects mediated, at least in part, by oxidative stress [8] and inflammation [9,10].

Although most prior telomere research has focused on adult populations, telomere attrition may be particularly prominent in early life, as shown in studies of childhood adversities [11]. Whether telomere length declines in very preterm infants before term equivalent age is largely unknown but is clinically relevant because these infants spend 2 to 4 months after birth in the neonatal intensive care unit (NICU), where numerous environmental exposures often lead to oxidative stress and inflammation-related illnesses such as chronic lung disease, sepsis, necrotizing enterocolitis, and retinopathy of prematurity. After discharge, preterm-born children are more likely to suffer from neurodevelopmental impairments [12–18] and asthma [19,20] than full term children, and as adults they are at higher risk for aging-related chronic diseases including chronic obstructive pulmonary disease [21], hypertension [22–24], and type II diabetes [25,26]. Additionally, a cross sectional study (n = 236) found an association between salivary telomere length and pulmonary function in adolescents born very preterm [27]. Consequently, some have suggested that preterm infants display an “aged” phenotype [28].

Telomere length is determined by both telomere length at birth (which serves as an “initial setting”) and by subsequent attrition [29]. Longitudinal data are limited regarding postnatal changes in telomere length during the NICU hospitalization [30]. Thus, the aim of our study was to determine the trajectory of telomere shortening during the NICU hospitalization in a contemporary cohort of preterm infants. We hypothesized that telomere length would measurably decline between birth and NICU discharge. We based this hypothesis on prior work indicating that preterm infants exhibit *longer* telomeres at birth [28,31], but have *shorter* telomeres in young adulthood compared with term-born infants [32], suggesting more rapid attrition early in life, and on two previous longitudinal studies of telomere length in the NICU [28,33].

## Materials and methods

We studied 10 infants born <32 weeks’ gestation at the Brigham and Women’s Hospital NICU (Boston, MA). The Partners Human Research Committee (FWA00000484) approved the study (protocol 2016P001020), and parents provided written informed consent. Patients were not involved in the design of this study.

Gestational age was defined by the best obstetrical estimate [34]. For 7/10 participants, the pregnancy dating was based on the first date of the last menstrual period (LMP) and confirmed by 1^st^ trimester ultrasound. Dating for the other 3 pregnancies was by LMP alone or in combination with 2^nd^ trimester ultrasound. We abstracted clinical data from the electronic medical record, including the variables needed to calculate the Score for Neonatal Acute Physiology, Perinatal Extension II (SNAPPE-II), a measure of neonatal illness severity [35]. Higher scores indicate greater illness severity. Postnatal diagnoses such as bronchopulmonary dysplasia were based on standardized definitions [36].

Dried blood spots were collected for research alongside clinically indicated blood draws such as state-mandated newborn screening tests, sealed with a desiccator, and stored at -80°C within 24 hours. We selected three blood spots per infant based on timing of collection within the first week (Time 1), approximately 1-month chronologic age (Time 2), and near discharge, which typically occurs around term equivalent age (Time 3) (**Table 1**).

**Table 1:** Participant characteristics (n = 10).

	Mean (SD) or n (%)	Range
Mode of delivery		
Vaginal	2 (20%)	
Cesarean	8 (80%)	
Antenatal steroids given	10 (100%)	
Gestational age at birth, w*eeks*	26.8 (1.7)	23.7, 29
Birth weight, *grams*	711 (201)	495, 1200
Birth weight percentile	18 (21)	0, 66
Male	6 (60%)	
Small for gestational age at birth	5 (50%)	
SNAPPE-II score	45.8 (24.7)	9, 86
Postnatal diagnoses		
Necrotizing enterocolitis	1 (10%)	
Bronchopulmonary dysplasia	7 (70%)	
Bacteremia	1 (10%)	
Chronologic age at blood sample collection, *days*		
Time 1	3.5 (1.6)	1, 6
Time 2	26.6 (4.9)	21, 37
Time 3	83.9 (22.0)	41, 116
Postmenstrual age at blood sample collection, *weeks*		
Time 1	27.3 (1.7)	24.3, 29.1
Time 2	30.6 (1.4)	27.6, 32.1
Time 3	38.8 (2.3)	34.1, 42.7

Birth weight percentile based on the Olsen reference [42]; small for gestational age defined as birth weight <10^th^ percentile. SNAPPE-II is the Score for Neonatal Acute Physiology Perinatal Extension II, a measure of neonatal illness severity [35].

We extracted DNA manually from six 3 mm punches using the QIAamp 96 DNA Blood Extraction Kit (Qiagen). DNA yields ranged from 14 to 329 ng. Additional extractions were performed for a few samples to increase the total yield above 40 ng. We measured relative telomere length using the Real-Time Quantitative PCR assay modified for a high-throughput 384-well format [37]. We assayed 5 ng genomic DNA in triplicate in either the Telomere or 36B4 (single-copy gene) PCR reaction mixture. Reagents used included Thermo Scientific Hyclone H_2_O, Applied Biosystems PowerUP SYBR Green Master Mix, Invitrogen Custom Primers (36B4d + 36B4u and Tel-1 + Tel-2), and DTT for Telomere Reaction. We used the Applied Biosystems QuantStudio 6 flex PCR machine. Telomere cycling conditions are listed in **S1 Table**.

We calculated the relative average telomere length as the ratio of the **T**elomere repeat copy number to a **S**ingle gene (36B4) copy number (T/S ratio) [37–41], reported as the exponentiated T/S ratio and corrected for a reference sample to account for plate-to-plate variation. A higher number indicates longer average telomere length. A relative average telomere length of 1.2 indicates that the experimental sample is 20% longer than the reference sample. The average coefficient of variation ([standard deviation/mean] x 100) in telomere length was 0.37%.

We described key characteristics of the sample using means and proportions. We then assessed within-individual change in relative telomere length using two approaches. We calculated the change in relative telomere length as the difference between measurements obtained at Time 3 and Time 1, which reflects the change that was experienced over the NICU hospitalization. Next, we used all three measurements to graph trajectories in telomere length by postmenstrual age (gestational age at birth plus postnatal age, in weeks) for each of the 10 infants via spaghetti plot. We chose to examine time as defined by postmenstrual age because this construct reflects both time in a developmental sense and time from birth. We used linear mixed effects models with random intercepts and slopes to assess the mean rate of change that infants experienced over time, adjusting for their gestational age at birth. This approach estimates within-individual change while accounting for infant-specific variation in both relative telomere lengths at their first assessment as well as the changes in telomere length experienced over time. We modeled repeated measures using compound symmetric covariance structure and modeled time as postmenstrual age, accounting for telomere length assessments occurring at variable intervals for each infant.

To explore the potential impact of infant status at birth on telomere length change, we conducted two sensitivity analyses. First, to account for neonatal illness severity, we further adjusted the regression model for SNAPPE-II score. Then, to examine possible differences by fetal growth status, we used stratified models to compare the change in telomere length observed among infants who were small for gestational age (SGA, defined as weight for gestational age <10^th^ percentile using the Olsen reference [42]) and not SGA. Statistical analyses were conducted using Stata MP 16.1.

## Results

**Table 1** shows participant characteristics and the timing of each telomere length measurement.

**Fig 1:**
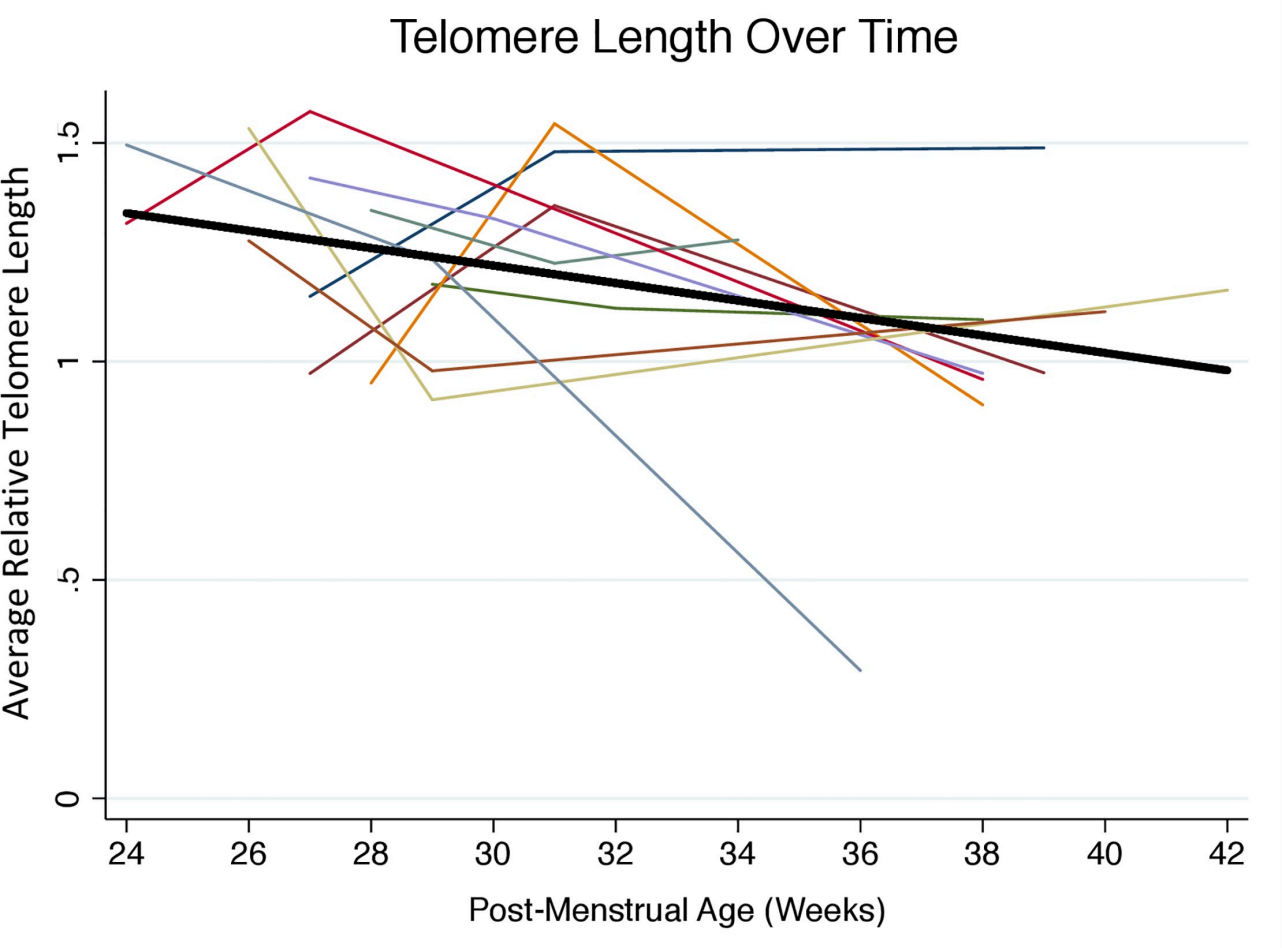
Change in relative telomere length with advancing postmenstrual age from birth to NICU discharge (n = 10, 3 measurements per participant). Relative telomere length was measured in triplicate by Real Time Quantitative PCR using DNA isolated from dried blood spots. Each participant is represented by a different color. The average decline in telomere length with advancing postmenstrual age and adjusted for gestational age at birth is represented by the solid black line, which has a slope of -0.02 units per week (95% CI -0.040, -0.0020; p = 0.03).

**Table 2** shows the longitudinal data on telomere length from all 10 infants, which is also presented graphically in **Fig 1**. Notably, in some instances, telomere length increased from an earlier to a later time point. The mean (SD) relative telomere length within the first week of hospitalization (Time 1) was 1.26 (0.2), and near discharge (Time 3) was 1.02 (0.3). On average, infants experienced a decline in telomere length by 0.24 units from week 1 to near discharge.

**Table 2:** Relative telomere length at 3 time points during the NICU hospitalization (n = 10).

	Relative telomere length, units/week		
Participant	Time 1	Time 2	Time 3
1	1.15	1.48	1.49
2	0.97	1.36	0.97
3	0.95	1.54	0.90
4	1.35	1.22	1.28
5	1.32	1.57	0.96
6	1.42	1.33	0.97
7	1.53	0.91	1.16
8	1.28	0.98	1.11
9	1.18	1.12	1.10
10	1.50	1.23	0.29
Mean (SD)	1.26 (0.2)	1.27 (0.2)	1.02 (0.3)

We detected little difference in relative telomere length according to gestational age at birth (0.004 units per week, 95% CI -0.040, 0.049, p = 0.8). Over time, relative telomere length declined by 0.021 per week of postmenstrual age (95% CI, -0.040, -0.0020, p = 0.03), adjusting for gestational age at birth (**Table 3**). **Fig 1** shows graphically this average decline in telomere length with advancing postmenstrual age, adjusted for gestational age at birth. Adjustment for neonatal illness severity minimally changed the estimate of telomere length change (**Table 3**). Sensitivity analyses excluding extreme observations also did not substantially change estimates (data not shown).

**Table 3:** Estimated decline in relative telomere length (units per postmenstrual week).

	N	Beta	Lower 95% CI	Upper 95% CI	P-value
Overall cohort, Model 1	10	**-0.021**	**-0.040**	**-0.0020**	**0.03**
Overall cohort, Model 2	10	**-0.021**	**-0.040**	**-0.0021**	**0.03**
SGA, Model 1*	5	-0.012	-0.039	0.015	0.39
Not SGA, Model 1*	5	**-0.031**	**-0.054**	**-0.0078**	**0.009**

Model 1 adjusted for gestational age at birth. Model 2 also adjusted for the Score for Neonatal Acute Physiology Perinatal Extension II, a measure of neonatal illness severity [35]. Higher illness severity defined as at or above the median score of 44. Birth weight percentile based on the Olsen reference [42]; small for gestational age (SGA) defined as birth weight <10^th^ percentile. Bold face type indicates p<0.05.

*Interaction p = 0.43.

Results of exploratory stratified analyses are shown in **Table 3**. Restricting to non-SGA infants, we observed a similar decline in telomere length as for the overall cohort, whereas the decline in SGA participants was close to the null (interaction p-value 0.43).

## Discussion

We present longitudinal data from a cohort of hospitalized preterm infants showing, as hypothesized, a decline in telomere length with advancing postmenstrual age from birth to NICU discharge. This observation is consistent with an Italian study of 46 very preterm infants [33], which found that telomere length declined by 0.04 units from birth to discharge using blood from different sources across follow-up (cord blood at birth, peripheral blood at discharge). By assessing peripheral blood only, our study minimized the measurement error that may result from examining blood from different sources. Our results indicate a steeper decline of 0.21 units over a similar time frame. The present findings are also consistent with a small (n = 5) study [28] of preterm infants that measured telomere length twice from peripheral blood and found that telomere length declined by 0.16 units between birth and term equivalent age (near NICU discharge). In our study, three repeated measures of telomere length allowed for a more accurate quantification of within-infant trajectory of telomere length change. Overall, our study adds to mounting evidence that telomere length declines on average during the NICU stay, which is noteworthy given infants’ relatively short duration of hospitalization (weeks to months). Additionally, our findings suggest that the rate of decline may be more rapid than previously reported.

Prior research highlights plausible biological pathways linking the NICU hospitalization with telomere attrition. Telomere loss early in life is accelerated by oxidative stress [8] and inflammation [9,10], both of which are well-documented in hospitalized preterm infants [43,44]. More frequent painful procedures–a source of stress in the NICU–have been linked with faster telomere attrition from birth to NICU discharge [45]. Additionally, exposure to potential toxins (e.g., phthalates in medical plastics) may also play a role [46,47]. Taken together, these findings led us to hypothesize that more rapid telomere attrition may occur postnatally in the NICU than would occur for a fetus who had remained in-utero.

In our study, the effect of aging in the NICU is reflected by the association of relative telomere length with advancing postmenstrual age (0.02 units per week). The ratio of the effect of aging in the NICU to the estimated effect of aging associated with increasing gestational age at birth (0.004 units per week) can provide an estimate of postnatal telomere attrition relative to estimated attrition occurring in-utero. Among infants in our sample, postnatal telomere attrition was 5 times faster than in-utero aging. Although our estimate of telomere shortening in relation to gestational age was limited by our sample size, this finding provides preliminary evidence that accelerated aging occurs in hospitalized preterm infants.

On average, telomere length declined over time among infants in our sample, but a few infants had longer telomere length measurements at their second assessment as compared to their first. This phenomenon has been described before. Emerging evidence suggests that temporary telomere lengthening may be common in early development. In a cohort of healthy children studied from infancy to 3 years of age, telomere lengthening from infancy to 2 years was noted in approximately 1/3 of the sample, and from 2 to 3 years of age in almost half of the sample [48]. In that study, telomere length was assessed in DNA extracted from saliva. Telomere lengthening has also been reported in young adults [49]. Possible explanations for observed increases in telomere length over time include measurement error, differences in cellular composition between samples, and unknown biological mechanisms [48].

Previous investigators examined whether telomere length at birth differs between SGA and non-SGA full term infants, and did not find a difference [50]. We explored whether SGA status at birth was associated with the rate of telomere change over time and found little evidence of a difference, but we had limited power to detect this or other possible interactions.

A strength of our study was the use of dried blood spots, which proved to be a highly feasible strategy for collecting repeated blood samples from small preterm infants in the NICU and yielded adequate quantities of DNA for measuring telomere length. A limitation of this approach is that we were unable to isolate white blood cells or subtypes. Collecting 3 repeated samples from each participant was a strength that enhanced statistical power. However, given our small sample size we were unable to examine the role of specific prenatal or NICU exposures or comorbidities on telomere length at birth or its change over time, nor were we able to investigate whether specific exposures such as oxidative stress and inflammation exerted immediate or latent effects on telomere length.

The early changes in telomere length that we observed among hospitalized preterm infants may have important clinical implications. Being born preterm is associated with an elevated risk of developing chronic disease later in life [51,52]. Although early clinical care can play a critical role in reducing the impact of preterm birth on early organ system development [53], the long-term outcomes associated with NICU care are challenging to evaluate because they require following children for years or decades after they leave the hospital. Telomere length at NICU discharge–or the extent of telomere shortening that occurs from birth to discharge–has the potential to serve as a predictive biomarker [54,55] of long-term health and development risk. Telomere length as a biomarker is potentially relevant for both clinical research and for the identification of infants at highest risk for later-life chronic conditions, so that personalized preventive strategies may be undertaken. Further investigation is required to determine the research applications and clinical utility of routinely assessing telomere length in the NICU population.

## Conclusion

Our findings suggest that preterm infants in the NICU experience measurable declines in telomere length. Future research should investigate clinical factors that influence telomere attrition rate in preterm-born infants and examine the utility of telomere length as a biomarker for identifying those infants at increased risk for future aging-related health problems.

## Supporting information

S1 Table Telomere cycling conditions. (DOCX)
