# Supplementary material for: Telomere length shortening in hospitalized preterm infants: A pilot study
Source: PLoS One. 2021 Jan 20;16(1):e0243468. doi: 10.1371/journal.pone.0243468 (PMC7817026; doi:10.1371/journal.pone.0243468)
Supplement: S1 Table — (DOCX) [file pone.0243468.s001.docx]

| **Supplemental Table 1. Telomere cycling conditions** |
| --- |
| Telomere Cycling: |
| HOLD: 50°C for 2 minutes               95°C for 2 minutes |
| PCR: 35 cycles as default. Run full 35 cycles for test plate and based on the plateau, you can change the # of cycle. |
| 95°C for 15 seconds |
| 54°C for 2 minutes    (Data Collection) |
| 36B4 Cycling: |
| HOLD: 50°C for 2 minutes               95°C for 2 minutes |
| PCR: 40 cycles as default. Run full 40 cycles for test plate and based on the plateau, you can change the # of cycle. |
| 95°C for 15 seconds |
| 58°C for 1 minute and 10 seconds (Data Collection) |
